# *Aster spathulifolius Maxim*. a leaf transcriptome provides an overall functional characterization, discovery of SSR marker and phylogeny analysis

**DOI:** 10.1371/journal.pone.0244132

**Published:** 2020-12-23

**Authors:** Sivagami Jean Claude, SeonJoo Park

**Affiliations:** Department of Life Sciences, Yeungnam University, Gyeongsan, Gyeongbuk, South Korea; University of Georgia, UNITED STATES

## Abstract

*Aster spathulifolius* Maxim. is belongs to the Asteraceae family, which is distributed only in Korea and Japan. The species is traditionally a medicinal plant and is economically valuable in the ornamental field. On the other hand, the *Aster* genus, among the Asteraceae family, lacks genomic resources and its molecular functions. Therefore, in our study the high-throughput RNA-sequencing transcriptome data of *A*. *spathulifolius* were obtained to identify the molecular functions and its characterization. The *de novo* assembly produced 98660 uniqueness with an N50 value of 1126bp. Total unigenes were procure to analyze the functional annotation against databases like non-redundant protein, Pfam, Uniprot, KEGG and Gene ontology. The overall percentage of functional annotation to the nr database (43.71%), uniprotein database (49.97%), Pfam (39.94%), KEGG (42.3%) and to GO (30.34%) were observed. Besides, 377 unigenes were found to be involved in the terpenoids pathway and 666 unigenes were actively engaged in other secondary metabolites synthesis, given that 261 unigenes were within phenylpropanoid pathway and 81 unigenes to flavonoid pathway. A further prediction of stress resistance (9,513) unigenes and transcriptional factor (3,027) unigenes in 53 types were vastly regulated in abiotic stress respectively in salt, heat, MAPK and hormone signal transduction pathway. This study discovered 29,692 SSR markers that assist the genotyping approaches and the genetic diversity perspectives. In addition, eight Asteraceae species as in-group together with one out-group were used to construct the phylogenetic relationship by employing their plastid genome and single-copy orthologs genes. Among 50 plastid protein-coding regions, *A*. *spathulifolius* is been closely related to *A*. *annua* and by 118 single copy orthologs genes, *O*. *taihangensis* is more neighboring species to *A*. *spathulifolius*. Apart from this, *A*. *spathulifolius* and *O*. *taihangensis*, genera have recently diverged from other species. Overall, this research gains new insights into transcriptome data by revealing and exposing the secondary metabolite compounds for drug development, the stress-related genes for producing resilient crops and an ortholog gene of *A*. *spathulifolius* for the robustness of phylogeny reconstruction among Asteraceae genera.

## Introduction

The *Aster* genus is a perennial flowering plant in the family of Asteraceae. There are 32,020 species contains 190 genera of which many of the asters are in the Astereae tribe, the second largest tribe [1]. The *A*. *spathulifolius* Maxim (seashore spatulate aster) is a diploid species belonging to the genus *Astersa*. It is an endemic plant occurring only in the coastal region of Korea and Japan. *A*. *spathulifolius* is widely distributed in Dokdo Island, which composed of volcanic rocks, pyroclastic sedimentary rocks and highly salinity environments, with smog and strong wind [2], and also it has been reported that the plants are difficult to grow under such challenging environmental conditions [3, 4]. The *A*. *spathulifolius* has high economic value, both in medicine and for ornamental purposes [5]. In medicinal perspectives, *A*. *spathulifolius* used to treat asthma and diuresis, and in human cancer cell lines with labdane diterpene compounds (secondary metabolites) [6]. Besides, *A*. *spathulifolius* was studied for antibacterial activity and carcinoma cell lines against oral pathogens [7] and its simple leaf extraction were used to treat hyperglycemia that inhibits the gluconeogenesis by accelerating glucose and lipid metabolism and helps against diabetes and diabetic complications [8, 9]. *In vivo* study on mice (diet-induced obesity) demonstrated, an anti-adipogenic, anti-lipogenic, anti-obesity and anti-hyperlipidemia effects [10, 11] in the manner of weight loss and fat mass reduction [12]. To date, the complete plastid genome with 13.6 kb inversion has been documented as genomics data for *A*. *spathulifolius* in NCBI [13]. In this current study, we used RNA-sequencing data to identify the functional genes involved in the leaf of *A*. *spathulifolius* for the first time. Moreover, the total unigenes were highly used to evaluate their multiple functional annotations on *A*. *spathulifolius*. The species was further elaborated on the production of different antibiotic and secondary metabolites for drug development. Furthermore, this study will be useful to identify stress-specified unigene, which support crops for better improvement. Phylogeny based on plastid protein-coding region and single-copy nuclear gene (orthologs gene) among Asteraceae genera along with *A*. *spathulifolius* were documented on this study.

## Materials and methods

### Plant material, RNA isolation and illumina sequencing

The whole plant of *A*. *spathulifolius* was collected from Dokdo Island (South Korea). The herbarium was prepared and deposited at Yeungnam University Herbarium (YNUH2018asp001) for future purposes. For the isolation of RNA, 100 mg of leaf tissue of the plant collected from the habitat condition (Dokdo island, SK) was used, and further preserved in liquid nitrogen and stored at—80°C. A total RNA isolation was performed following the protocol of Breitler *et al*. [14]. Using DNase 1 (Invitrogen DNase 1 Amplification kit, USA) treatment to obtain purified RNA from DNA contamination. The total RNA (47.50 μg) were used to sequence via Illumina HiSeq 2000 platform at Lab Genomics (Seongnam, South Korea), to generate total 21 GB reads of 151 bp of paired-end library preparation, the TruSeq standard mRNA kit was used for library preparation.

### *De novo* assembly, alignment and transcript quantification

The paired-end raw reads were quality assessed by FastQC v0.11.218. The raw paired-end reads were quality assessed by FastQC v0.11.218. Phred score > = 30 reads were retained using Trimmomatic tool to trim reads below 30% quality from the 148035505 raw reads. The filtered reads was further used for transcriptome assembly using Trinity v2.8.2 tool [15]. Trinity, a *de novo* assembler tool based on sequence data into many individual de Bruijn graphs with default k-mer size (k = 25) and which followed by Inchworm is to generate full-length isoforms from raw reads, Chrysalis cluster the contig which was generated by Butterfly modules to generate full-length transcripts or unigenes. Later Transdecoder v5.5.0 tool was used to retain peptide sequences for further analysis of blast. The Tophat v2.1.0 –Bowtie v2.3.5.1 alignment was used to align the raw reads to the retained transcripts [16, 17]. Estimation of abundance unigene was determined by RSEM v1.3.1(RNA-Seq by Expectation-Maximization) tool [18], which first generates and processes of total transcripts and then aligns to raw reads of *A*. *spathulifolius*. RSEM is used calculate the fragments per kilobase per million (FPKM) and transcripts per million (TPM) values of total unigenes to analysis the maximum number of gene expressed or abundant in *A*. *spathulifolius*. The both FPKM and TPM values are calculated to estimate the expression levels of unigenes involved in phenylpropanoid pathway (PPP) and caffeoyl quinic acid production of secondary metabolites.

### Functional annotation and analysis

The *de novo* assembled sequences of *A*. *spathulifolius* were used to identify the most significant match against the National Centre for Biotechnology Information (NCBI) database like the non-redundant protein (nr) and nucleotide using BLASTX tool with an E-value (1E-05). The BLASTP tool used against the Uniprot database (https://www.uniprot.org/) was downloaded for uniprotein identification with E-vaue of 1E-05. Pfam related unigene was retained using HMMER. The nr, Pfam and Uniprot database results are further used to retrieve Gene Ontology (GO) terms. The retrieved GO terms were classified into three categories: Cellular Component, Molecular Function and Biological process. Further, KEGG Automated Annotation Server (KAAS) was used for pathway mapping of species specified data with 1E-05 parameter.

### Transcription factor identification and stress gene finder

The transcriptional factor unigenes were identified using http://planttfdb.cbi.pku.edu.cn/ tool. The identified TFs were uploaded to obtain the Gene ontology function. The stress-related unigenes were estimated using the TRAPID program web tool (http://bioinformatics.psb.ugent.be/webtools/trapid/) and BlastX (TAIR database). The parameter used was 1E-05 to both the transcriptional factor and TRAPID analysis. Further confirmation of stress resistance genes by BlastP against the non-redundant proteins and the Uniprotein database has been performed.

### Identification of SSRs marker

The MicroSAtellite identification tool (MISA) was used to identify the simple sequence repeats marker from the total assembled unigenes of *A*. *sphathulifolius* [19]. The parameters were set to identify perfect repeat nucleotide motifs with a minimum threshold of (1/10) (2/6) (3/5) (4/5) (5/5), and (6/5).

### Phylogenetic analysis of plastid and nuclear gene

To determine the Aster group phylogenetic relationship with other known Asteraceae species (S1 Table), 50-plastid protein-coding region was chosen from the entire plastid genome. The plastid genomes of these species were retrieved from NCBI database. MAFFT was used to align the 50-plastid protein-coding region and then aligned sequences were loaded to the fast tree to construct the phylogeny tree among nine species. For the plastid-based phylogeny and visualization, the GeneiousR11 version was used. Transcriptome data for orthologs genes were used to draw the phylogenetic relationship between the eight species from Asteraceae as in-groups and one out-group from Goodeniaceae family. The SRA raw database from NCBI was downloaded using the SRA tool kit and fastq-dumb tool to extract the leaf and right raw reads, *de novo* assembly done by Trinity with Trimmomatic tool and Transdecoder to find ORF (S2 Table). The longest ORF coding region was extracted from all contig to minimize the layoff. Then, the cdhit tool [20] was used to reduce the redundancy of amino acids. Orthofinder [21] tool were finally ran to analyze the single-copy orthologs genes (auto selection of out-group) to construct phylogeny tree.

## Results and discussions

In total, 148035505 raw reads were generated from *A*. *spathulifolius* leaf transcriptome that accounts for approximately 21 GB of paired-end sequencing data and in that, 40.49% of the GC content were retained. The raw data were deposited at National Centre for Biotechnology Information (NCBI) Short Read Archive (SRA) database under the accession number SRR10724565. Overall, 163,022 assembled transcripts were generated with an average size of 908 bp; 98,660 unigenes with an average length of 722.83 bp and an N50 contig length of 1,126 bp. Read mapping of the raw reads showed that 89.1% were aligned by Tophat. The *De novo* based transcriptome-assembled details are given in Table 1.

**Table 1:** Statistical information of *de novo* assembly of *A*. *spathulifolius* leaf.

Data Information	Stats
Number of raw reads	148035505
GC content	40.49%
Total transcripts	163,022
Total Unigene	98,860
Median contig length	425
smallest contig	207
largest contig	11,859
contig N50	1,412
Mapping to the raw data	89.91%

### Functional annotation of unigenes

**Fig 1:**
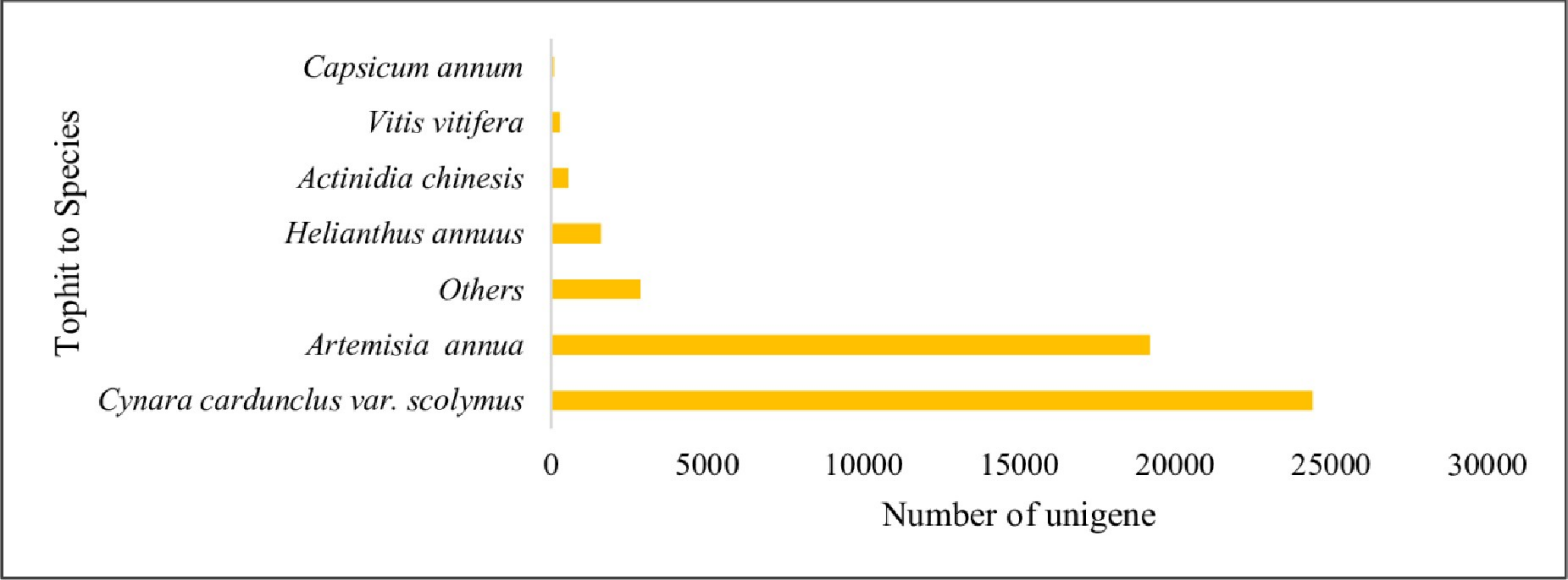
Tophit to the species of nr database by blastP.

The total unigenes of *A*. *sphathulifolius* annotated using blastP analysis against the non-redundant protein database showed 43,221 unigenes (43.71%) with an E-value cut of 1E-05. The unigenes showed top-hit species similarity with *Cynara cardunculus* var *scolymus* (24.41%), *Artemisia annua* (19.20%) and *Helianthus* (1.3%) and others (Fig 1). The results indicated that *Cynara* is more associate to *A*. *spathulifolius*. The annotations against proteins in the Pfam database showed 39,890 (39.94%) significant hits. Moreover, 46,869(49.97%) unigenes to the Uniprot, 29,880 (30.1%) to the GO classification, and 41,776 (42.34%) to the KEGG-KASS were annotated (Table 2). Indeed, there are unidentified unigenes are not known in this study due to no full discovery of differential function to the Asteraceae species. There are numerous unigenes are not identified through NCBI database though, this might number of Non-coding genes also included, which play great role in controlling the expression of transcriptome products [22].

**Table 2:** Functional annotation of different database and percentage of unigene annotated.

Database	Unigene	Percentage
Nr	47,282	47.80%
InterPro	54,781	55.55%
Pfam	39,890	39.94%
GO	29,921	30.34%
Uniprot	46,869	49.97%
KEGG	41,776	42.3%

### Functional classification of unigenes

**Fig 2:**
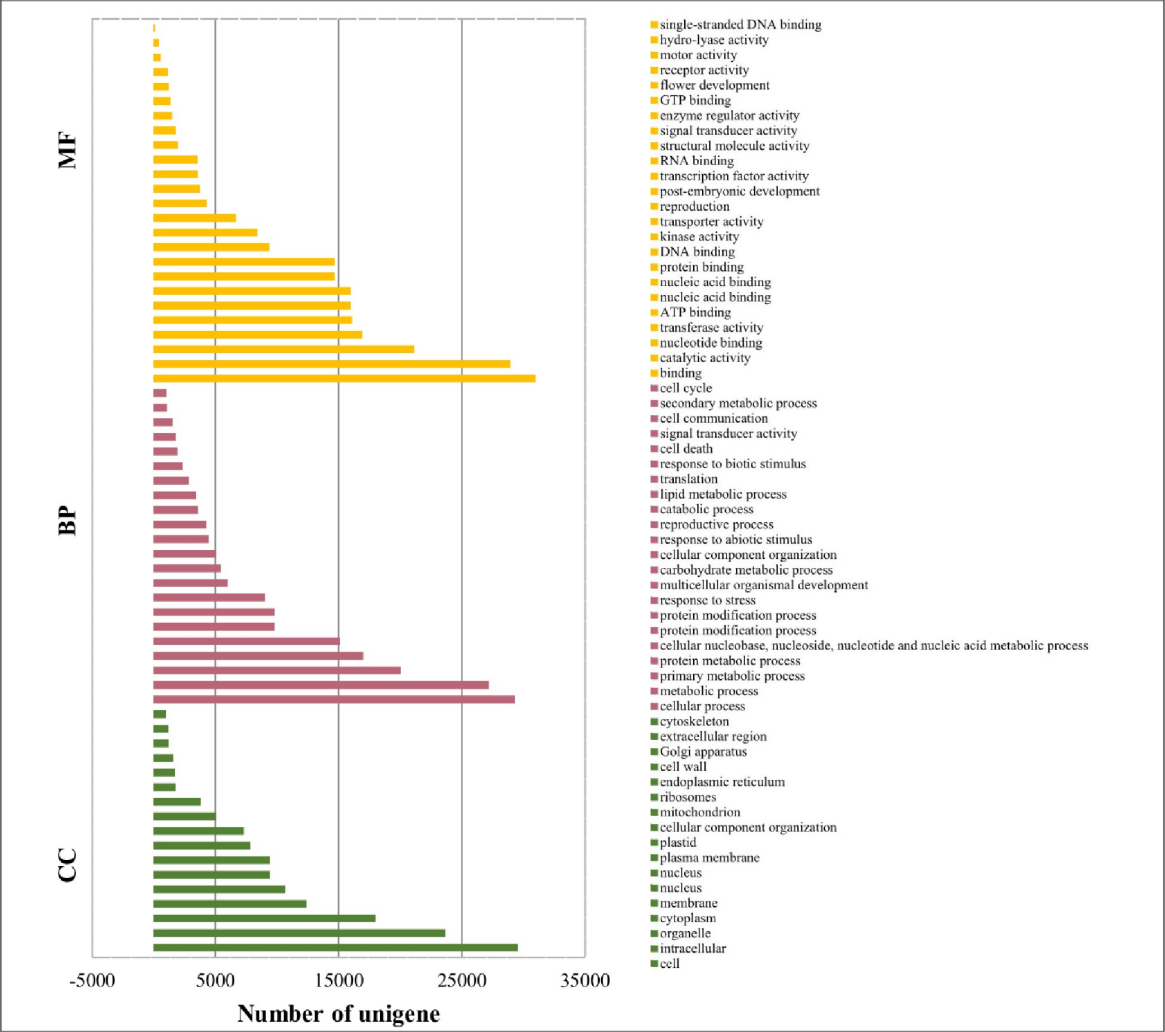
Gene ontology against *A*. *spathulifolius*: Top 60 enriched GO classification.

The Gene Ontology classification was retrieved based on the annotated unigene to the nr, Pfam, InterPro, and Uniprot databases. In total, 28,621 unigenes were assigned to 64 classes. In terms of the biological functions, there were 9,206 unigene involved, in which 4,447 responded to the abiotic stimulus, 9,823 to the protein modification process and 9024 responded to the stress. To molecular functional category, 12,124 unigenes were assigned to 26 classes involving, reproduction (4,288), post-embryonic development (3,755) and flower development (1,229). In the cellular components, there were 8,894 unigenes categorized and grouped into 27classes, where 5,085 unigenes were classified into the cellular component organization. (Fig 2).

### KEGG annotation

**Fig 3:**
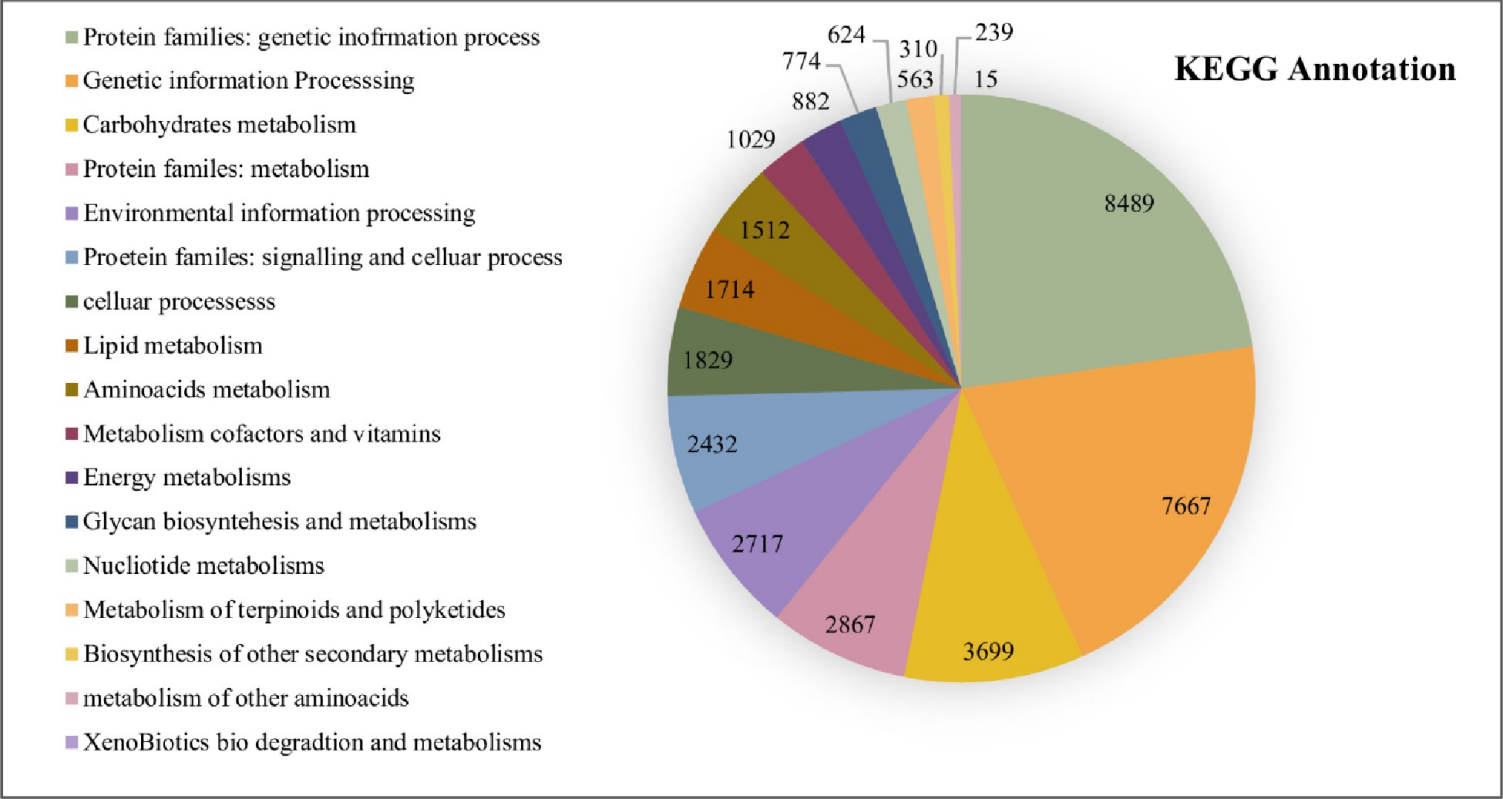
KEGG-KASS annotation to the *A*. *spathulifolius*.

**Fig 4:**
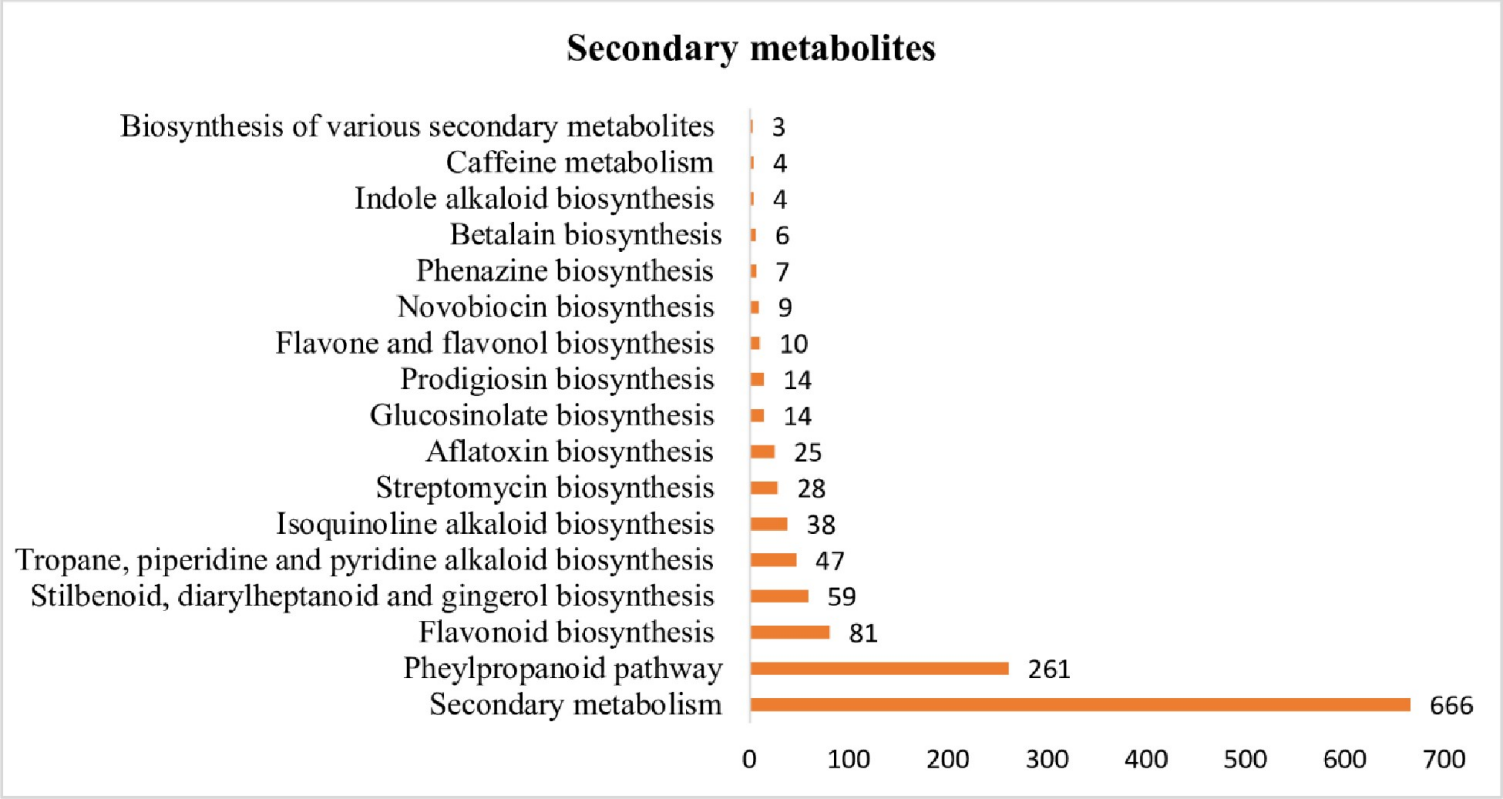
Biosynthesis of secondary metabolites pathway by KEGG-KAAS.

**Fig 5:**
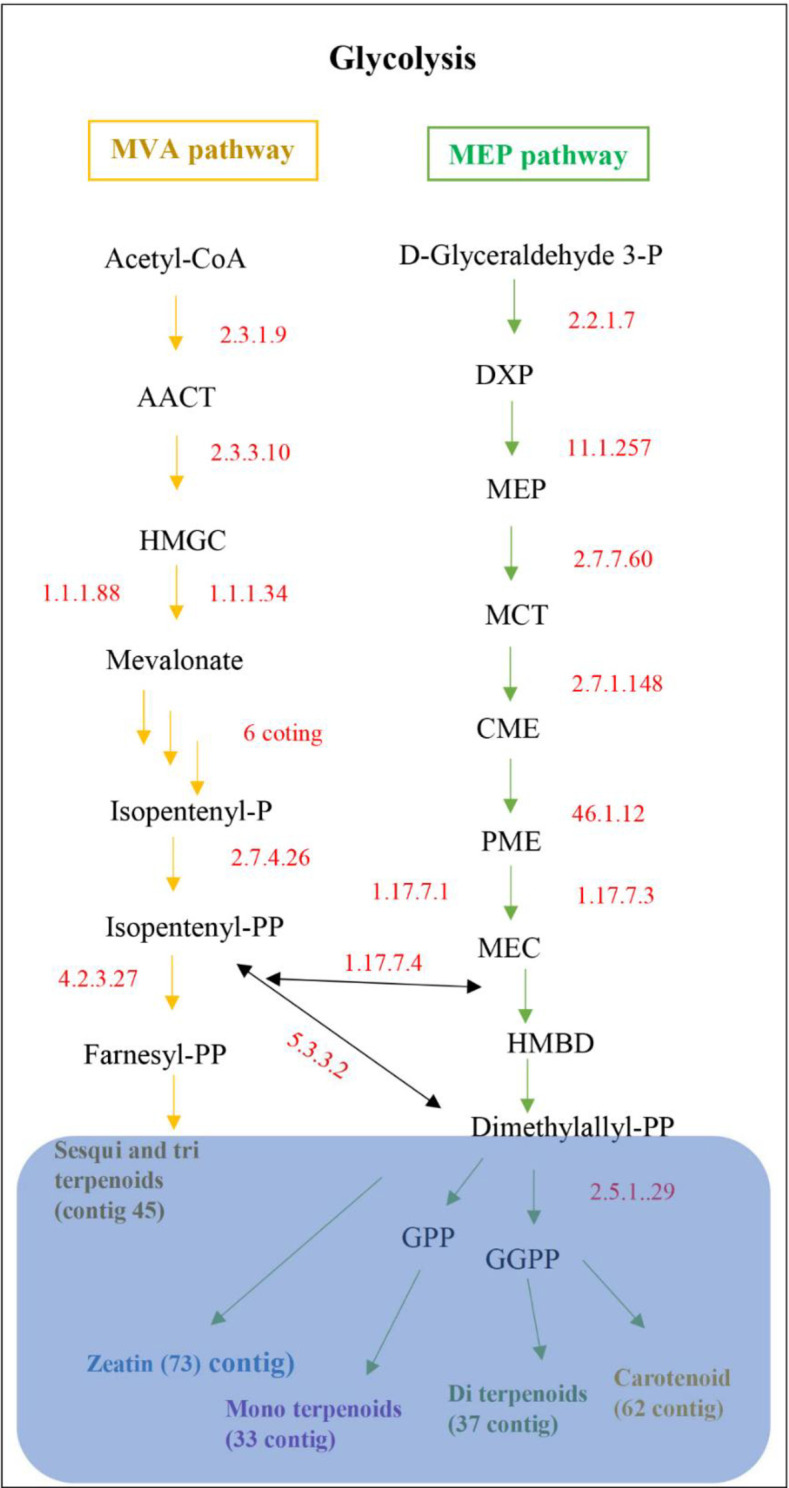
KEGG-KAAS analysis depicting 377 unigenes involved in MVA and MEP pathway and their secondary metabolism products from *A*. *spathulifolius* maxim. Down arrow marks indicates steps of enzymatic reaction involved.

**Fig 6:**
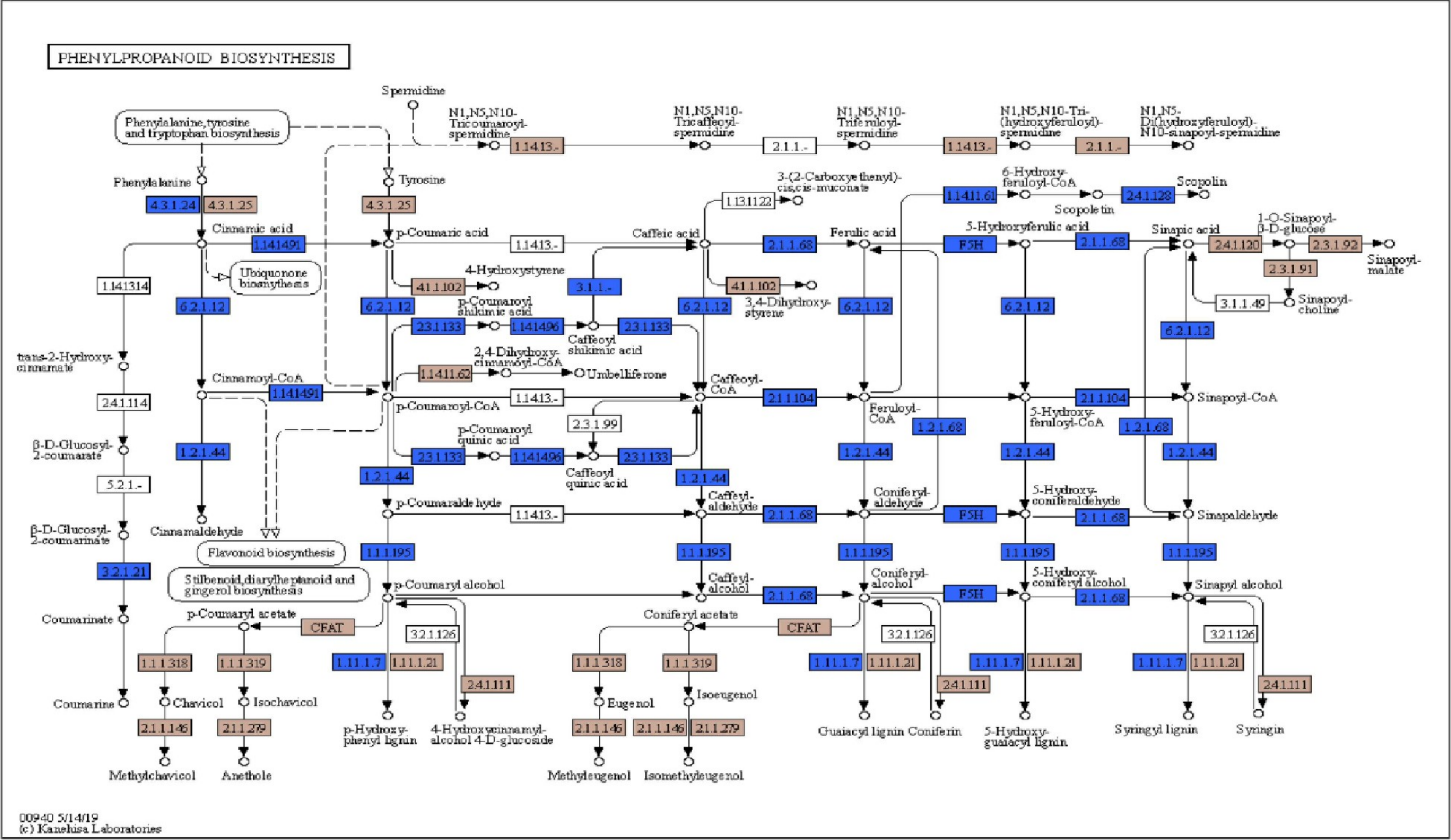
KEGG annotation showing unigenes involved in phenylpropanoid biosynthesis of *A*. *spathulifolius*.

Total unigenes of *A*. *spathulifolius* was performed using KAAS and KEGG map orientated database to reveal the molecular interaction networks and metabolic pathways. KEGG-KAAS analysis revealed 41,776 (42.34%) unigenes mapped to the different types of metabolic functions (Fig 3). 666 unigenes involved in the biosynthesis of secondary metabolisms, and 201 in the biosynthesis of antibiotics, respectively, which indicates that *A*. *spathulifolius* has an abundant of therapeutic benefit compounds, as previously stated elsewhere (Fig 4) [6–9]. In the terpenoid pathways, 377 unigenes are mapped and are classified as organic compounds that include terpenes, diterpene, and sesquiterpene. *A*. *spathulifolius* highly regulates the terpenoids production, such as mono, di, carotenoid, and sesqui and triterpenoids (Fig 5). In particular, 81 unigenes were involved to the flavonoid synthesis, and 261 unigenes were strongly expressed in phenylpropanoid pathway (PPP) and caffeoylquinic acid production (CQA; is a major chlorogenic acid presents in herbs, fruits, and vegetables). Previously many reports were highly focused on phenylpropanoid synthesis (Fig 6), to help in the reduction of lipid accumulation and adipogenesis [9–12, 23]. Therefore, we used RSEM to estimate the significance amounts of abundant unigenes among some of the PPP involved unigenes. RSEM are estimate to calculate the TPM and FPKM values are determines the expression that result in highly regulated and biosynthesized of PPP by-products in *A*. *spathulifolius*. In phenylpropanoid pathway, the high TPM values for Phenylalanine Ammonia Lyase (PAL) was 840648.07, which is main key product of PPP pathway that leads to different by-product throughout the PPP metabolites. For caffeic acid 3-O-methyltransferase that leads to synthesis of CQA was 44571.09, which is first product of PPP pathway that leads to different by-product throughout the secondary metabolites. In addition, cinnamoyl-CoA reductase was 39204.2 respectively and so on (S1 Table) this indicates the transcription frequency of a specific unigenes expression in *A*. *spathulifolius*. The most abundant unigenes for *de novo* assembled *A*. *spathulifolius* transcriptome expected to have gene abundantly in many pathway like the one flavonoids also observed. Furthermore, 137 unigenes were involved in the alpha-linolenic acid metabolism and 60 unigenes for in linoleic acid—lipid metabolism, which could not be produced by mammals (S1 Fig). In addition, 54 unigenes were mapped in the folate biosynthesis (Vitamin B9), which plays an important role in DNA replication and cell division when the organism is exposed to stress conditions. As a result, it can be noted that *A*. *spathulifolius* has a stress-tolerant function that helps plants grow in harsh environments. 330 unigenes were involved in the thermogenesis process (oxidative phosphorylation) in the mitochondria cell and have assisted in a high-fat diet-induced process [10, 23, 24]. This study is useful for the pharmaceutical industry in the development of drugs in future.

### Stress resistance gene and transcriptional factor

**Fig 7:**
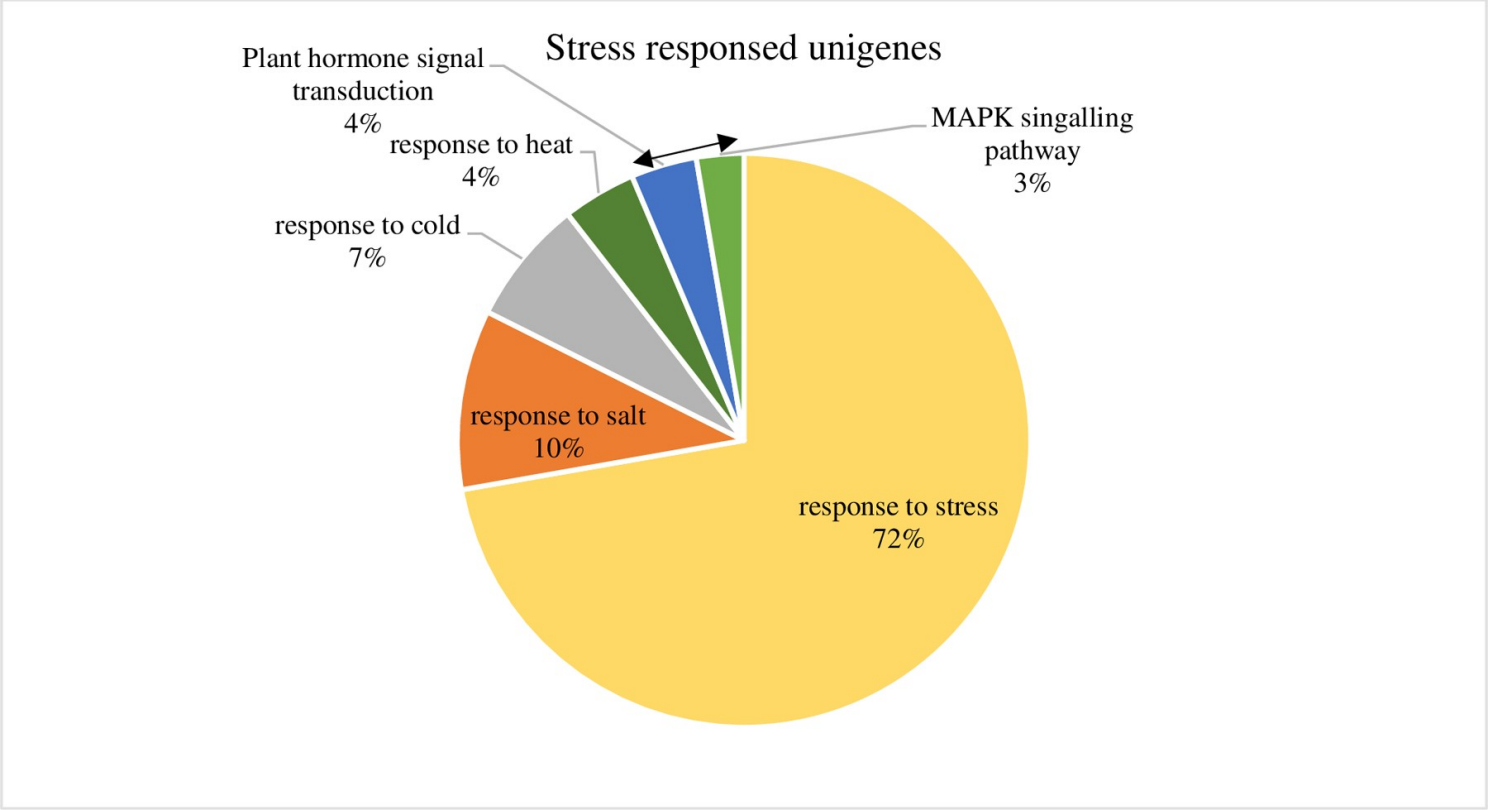
Unigenes expressed to stress related gene in Blastx (1E-05), KEGG and blast tool. Double side arrow mark indicates KEGG analyzed unigenes to the stress response.

**Fig 8:**
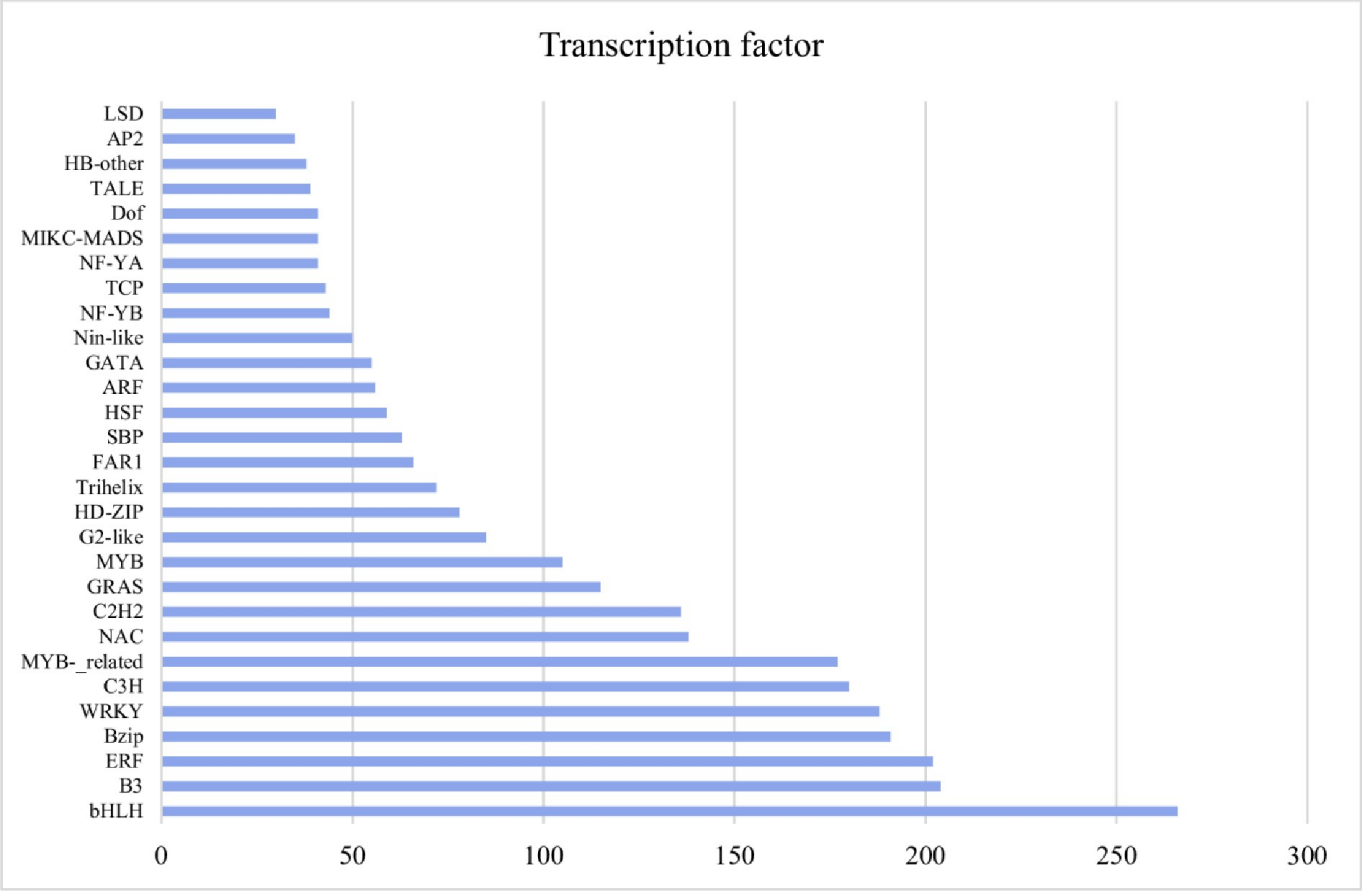
Top 30 enriched transcription factors unigenes in *A*. *spathulifolius* at 1E-05 parameter.

In nature, the plants are ultimately exposed to different stress factors, such as salt, cold, drought and heat that limit plant growth and productivity. As a result, we found 9,513 unigenes related to stress. 9,024 unigenes were retrieved from *Arabidopsis thaliana* databases, in which 1335 responded to the salt, 932 to the cold, and 547 to the heat and in addition others stress response unigenes were observed (Fig 7). Recently, Baillo et al. [25] stated that Transcriptional factor genes are highly involved in biotic and abiotic stress and are excellent candidates for crop improvement [26]. The present study revealed 53 types of transcriptional factors by using TF-plant tool (Fig 8). 3,167 unigenes were hit to the reference species of *Arabidopsis thaliana*. The highest number was found to be bHLH (basic helix-loop-helix) type, which is involved in drought stress [27]. The B3 ARF (Auxin response factors), ABI3 (Abscisic acid Insensitive3) and RAV (Related to AB13/VP1)) type were the second most involved in dehydration [28] and heat stress [29]. Followed with the ethylene response factor (ERF), which plays a vital role in extreme heat, cold, salt, and drought, and ultimately helps plant to grow under any stress environment [30]. In addition, the bzip (Basic Leucine Zipper) type responds to the binding onto the promoter region and controlling the expression of the gene [31]. WRKY (WRKYGQK motif along with zinc-finger) responds to both biotic and abiotic responses [32] and C3H, which play a role in salt responses [33]. These transcription factors strongly regulates the stress-responsive genes in *A*. *spathulifolius* and thus normalize the plant growth in its regular life cycle.

### Stress response unigenes in KEGG annotation

In total, 489 unigenes were observed from the KEGG annotated pathway of plant hormone signal transduction (Fig 8). As a result, an abundance of stress genes indicates the harsh environments that promote plants to produce stress-related unigenes. 352 unigenes were involved in the MAPK-signaling pathway (S2 Fig), where mitogen-activated protein kinase (MAPK) cascade plays a powerful role in biotic and abiotic stress regulating in plants [34]. 62 unigenes were mapped to the salt and cold tolerance pathway, corresponding to pathogen attacks, heavy metals, salinity, and drought conditions (S3 Fig). In addition, 402 unigenes were involved in plant-pathogen interaction [35].

### SSR identification

**Fig 9:**
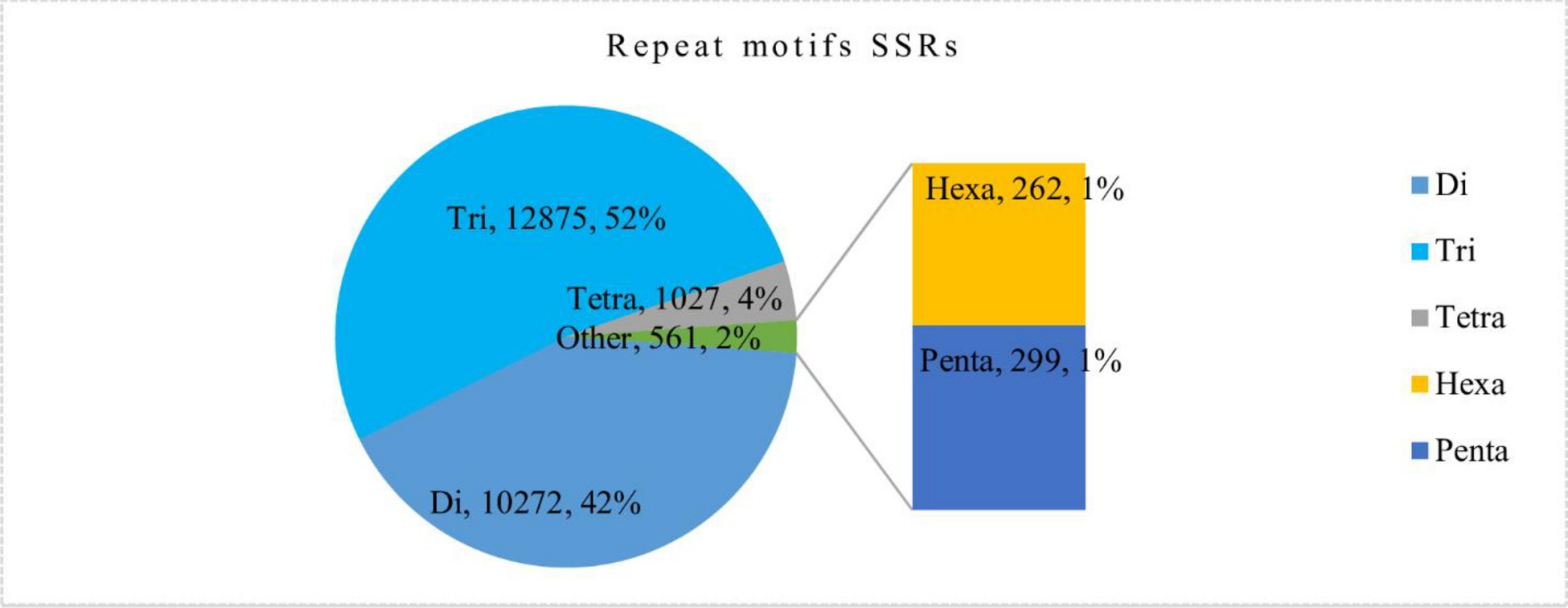
Distribution of repeats motifs of SSR in *A*. *spathulifolius*.

**Fig 10:**
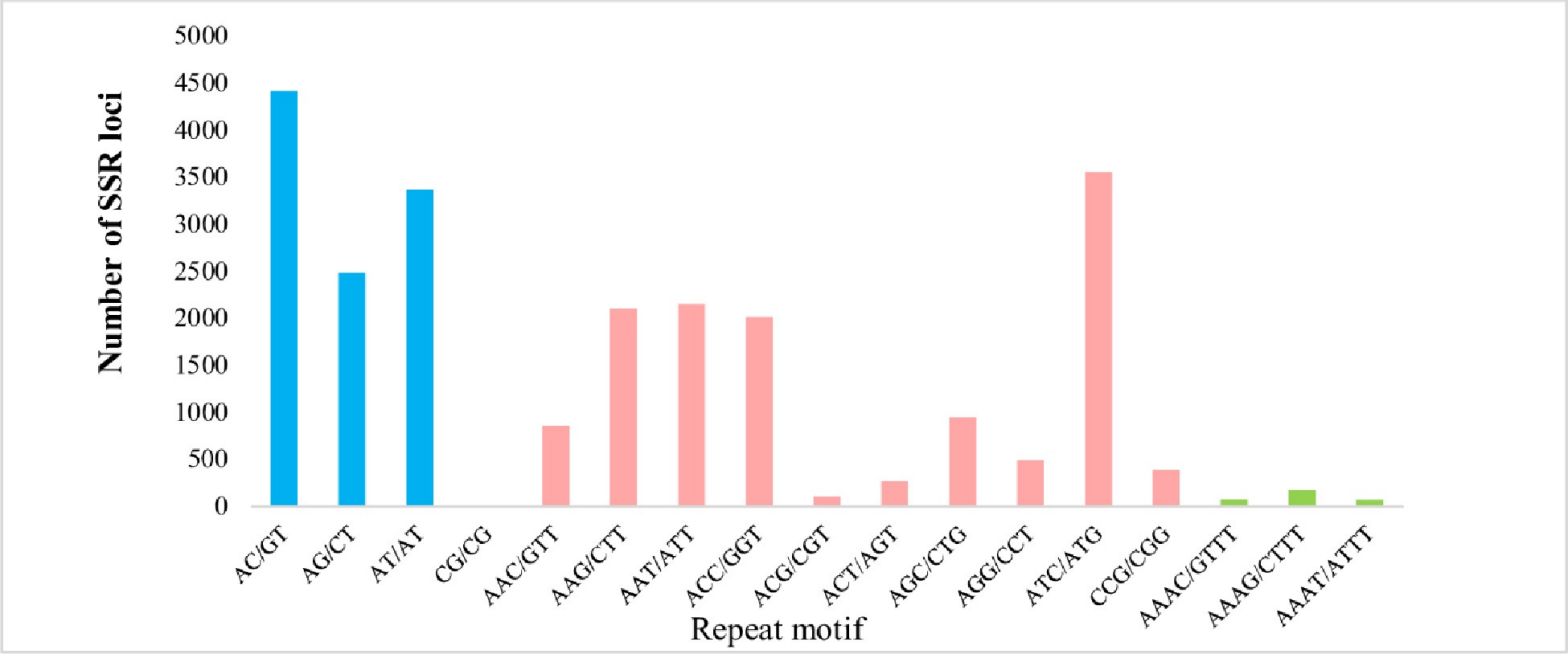
Distribution of repeats motifs of base in *A*. *spathulifolius*.

Microsatellites marker are profusely present in the DNA genome. SSR markers are beneficial for research on genetic diversity, population genetics, genotype, plant breeding improvement, and genetic linkage analysis. Presently, the SSR marker is very cost-effective and well determines the genetic diversity among population species studies based on transcriptome data and studied previously in *Chrysanthemum indicum* based on intraspecific genetic divergence [36, 37]. Out of 163,022 assembled transcripts from *A*. *sphathulifolius*, 38,545-SSRs markers were identified, which included 3,907 SSRs in the compound formation and 29,698 containing SSRs with an additional 6,896 sequences, showing more than one SSR (Fig 9). Mostly tri-nucleotide repeats with 12,875 (52.05%) repeat motifs were found and followed by di-nucleotide repeats 10,272 (41.52%), tetra-nucleotide 1,027 (4.15%), penta-nucleotide 262 (1.05%) and hexa-nucleotide 299 (1.20%). SSRs with five tandem repeats (6,772) were most common in *A*. *spathulifolius* followed by six repeats (6,285), and seven repeats (3,628), eight repeats (2,454), nine repeats (1,269) and ten repeats (6,923). Among di-nucleotide repeats, AC/GT (18.68%) followed by AG/CT (10.49%) and AT/AT (14%). AAC/GTT (3.63%) showed the highest frequency of Tri-nucleotide repeats motifs followed by AAG/CTT (9.98%), ATC/ATG (15.04%), AGC/CTG (4.09%) and other motifs, which were uniformly distributed (Fig 10).

### Phylogeny analysis of *A*. *spathulifolius*

**Fig 11:**
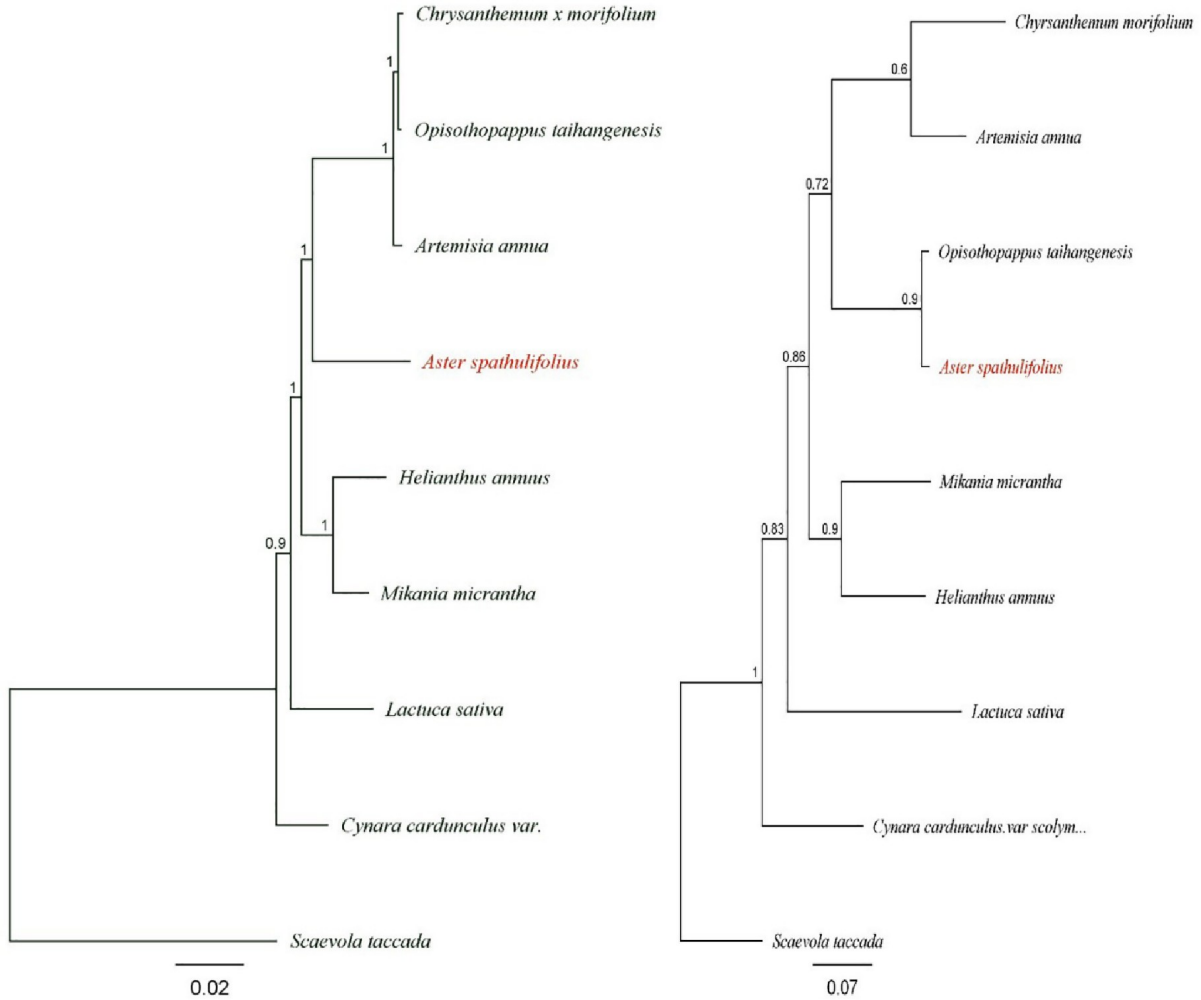
Phylogenetic relationships of major clades of Asteraceae phylogeny of plastid and single-copy orthologous genes. a) Maximum likelihood divergence, estimation tree shows the *A*. *spathulifolius* relationship of plastid genome protein-coding region. b) Divergence estimation of orthologous genes of nine species using Orthofinder and scale bar indicates the substitution rate.

The maternal gene-based phylogeny was constructed to examine the relationship between *A*. *spathulifolius* with eight in-group Asteraceae species such as *Artemisia*, *Chrysanthemum*, *Helianthus*, *Lactuca*, *Opisthopappus*, *Cynara*, *Mikrania* and one out-group namely *Scaevola* from the Goodeniaceae family. Based on the nucleotide alignment of 50 plastid protein-coding genes, the phylogenetic tree had a likelihood score >98% bootstrap value among the Asteraceae species. Plastid protein-coding phylogeny tree showed that *A*. *spathulifolius* forms one clade to *A*. *annua*, *O*. *taihangensis* and *C*. *x morifolium* lineage. To construct, single-copy orthologs genes based phylogeny complete ORF unigenes were retrieved from nine species (S2 Table). *A*. *spathulifolius* resulting to close affiliate with *O*. *taihangensis*, *A*. *annua*, and *C*. *x morifolium* in one clade with 89.80% bootstrap value than in the plastid protein-coding gene (Fig 11A). *C*. *cardunculus*.*var*. Scolymus and *L*. *sativa* were chosen as outgroup among other genera. Especially, in single-copy orthologs gene based phylogeny, *A*. *spathulifolius* found to be in one clade with *O*. *taihangensis* (Fig 11B), whereas, plastid protein-coding genes to *A*. *annua*. This result indicates that their phylogeny tree relationship varies due to past and high divergence in the nuclear gene than the plastid protein-coding gene and ultimately similar to the previous reports where *Cynara* and *Lactuca* were ancestors to the *Aster*, *Chrysanthemum*, *Helianthus* and to other genera [38]. Based on gene trees, first duplication event among Asteraceae genera (S4 Fig) shows that without out-group (*S*. *taccada*) *Cynara* gives rise to *Lactuca* and then the second duplication takes place to form the two new clades, of which, one is *Mikrania* and *Helianthus* (N4) branch. In other hand, *Artemisia* and *Chrysanthemum* (N5) branch followed by *Aster* and *Opisthopappus* (N6) genera. This pattern argues *Aster* and *Opisthopappus*, a recent divergence from other genera. Nevertheless, the more transcriptome species data will be needed to interpret a wide evolutionary relationship among the complete Asteraceae genera.

## Conclusion

The main aim of our study was to analyze the species of the genus *Aster* (*A*. *spathulifolius*) species that has not yet been extensively studied. The transcriptome of *A*. *spathulifolius* leaf using Illumina high throughput RNA sequencing platform were used. The identification of the uni-transcripts involved in molecular function, classification, and phylogenetic analysis was characterized. In this study, KEGG pathway analysis revealed the number of unigenes involved in flavonoid biosynthesis and highly expressed in *A*. *spathulifolius* leaf transcriptome. Medicinally significant aromatic amino acids (AAAs), especially high in phenylalanine compounds were found rich in *A*. *spathulifolius* regulating the Chlorogenic acid (CGA) biosynthesis. The thermogenesis process may increase by the CGA production that alters glucose and lipid metabolism. Furthermore, the *A*. *spathulifolius* plant, which grows in hostile environments, is prone to produce generous amounts of stress response unigenes to combat abiotic stress. That concludes the *A*. *spathulifolius* is more resistant to abiotic stress (heat, salt, drought, and others). Likewise, to estimate the divergence of *A*. *spathulifolius*, phylogeny of Asteraceae species using a single-copy orthologs gene from transcriptome data was constructed and studied. As a result, researching medically essential and stress-resistance plants like *A*. *spathulifolius* is a dynamic resource for the future.

## Supporting information

S1 Fig KEGG annotation to the alpha- linolenic acid metabolism in *A*. *spathulifolius*. (DOCX)

S2 Fig MAPK- signaling pathway mapped to the KEGG annotation against *A*. *spathulifolius*. (DOCX)

S3 Fig Plant hormone signaling pathway in *A*. *spathulifolius* against KEGG annotation. (DOCX)

S4 Fig Orthofinder: Gene duplication prediction among the Asteraceae family, N1-N6 indicates the duplication of gene in order, AAN: *A*. *annua*, OTA: *O*. *taihangensis*, ASP: *A*. *spathulifolius*, HAN: *H*. *annuus*, MMI: *M*. *micranta*, LSA: *L*. *sativa*, CCA: *C*. *cardunculus*. (DOCX)

S1 Table RSEM calculate expression matrix show main gene involved in phenylpropanoid pathway at FPKM rate. (DOCX)

S2 Table NCBI plastid and SRA database accession number along with information of assembled unigene in total. (DOCX)
