# Supplementary figures and images for: Aster spathulifolius Maxim. a leaf transcriptome provides an overall functional characterization, discovery of SSR marker and phylogeny analysis
Source: PLoS One. 2020 Dec 23;15(12):e0244132. doi: 10.1371/journal.pone.0244132 (PMC7757906; doi:10.1371/journal.pone.0244132)

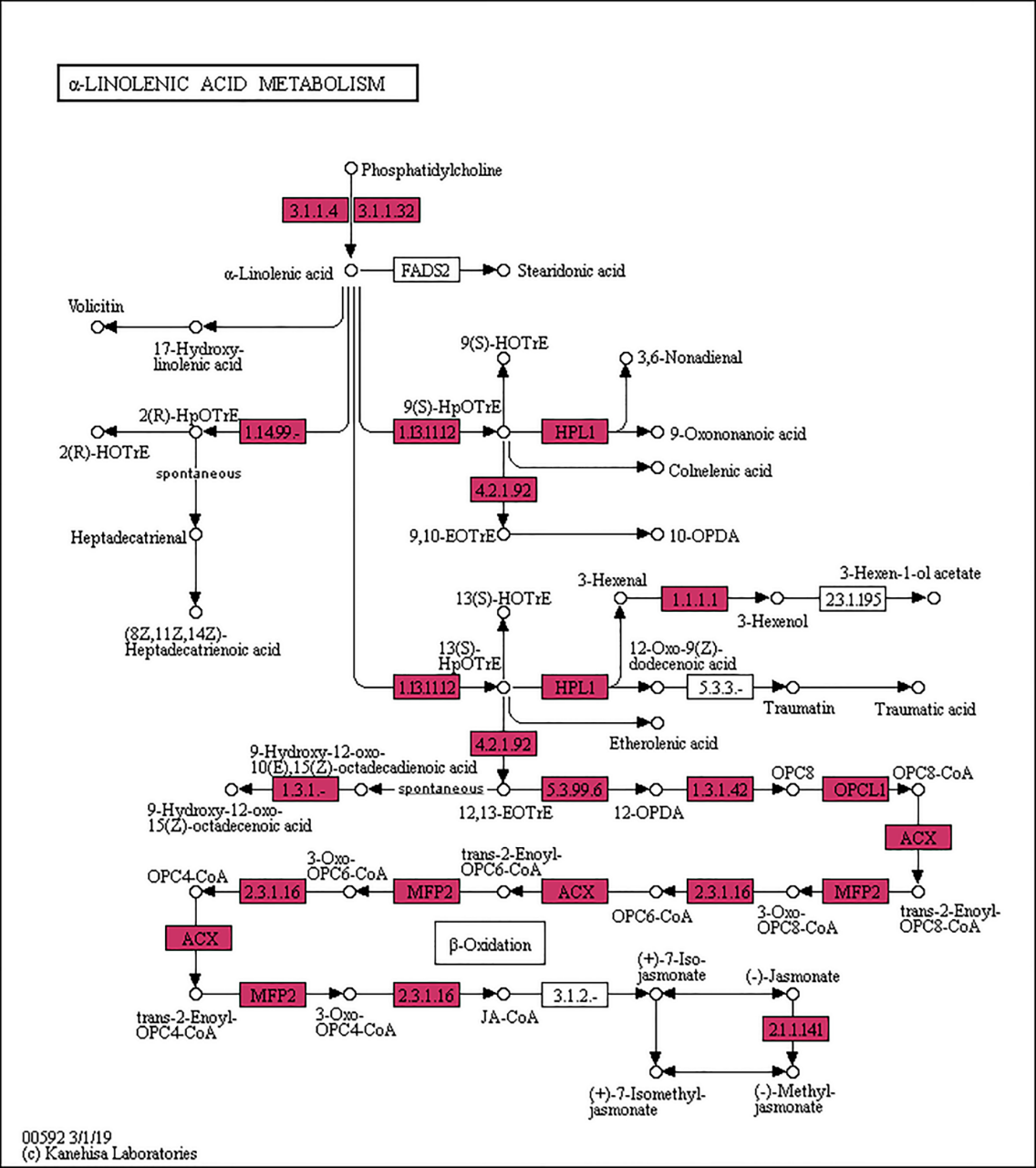


S1 Fig. KEGG annotation to the alpha- linolenic acid metabolism in *A. spathulifolius*

Supplement: S1 Fig — (DOCX) [file pone.0244132.s001.docx]

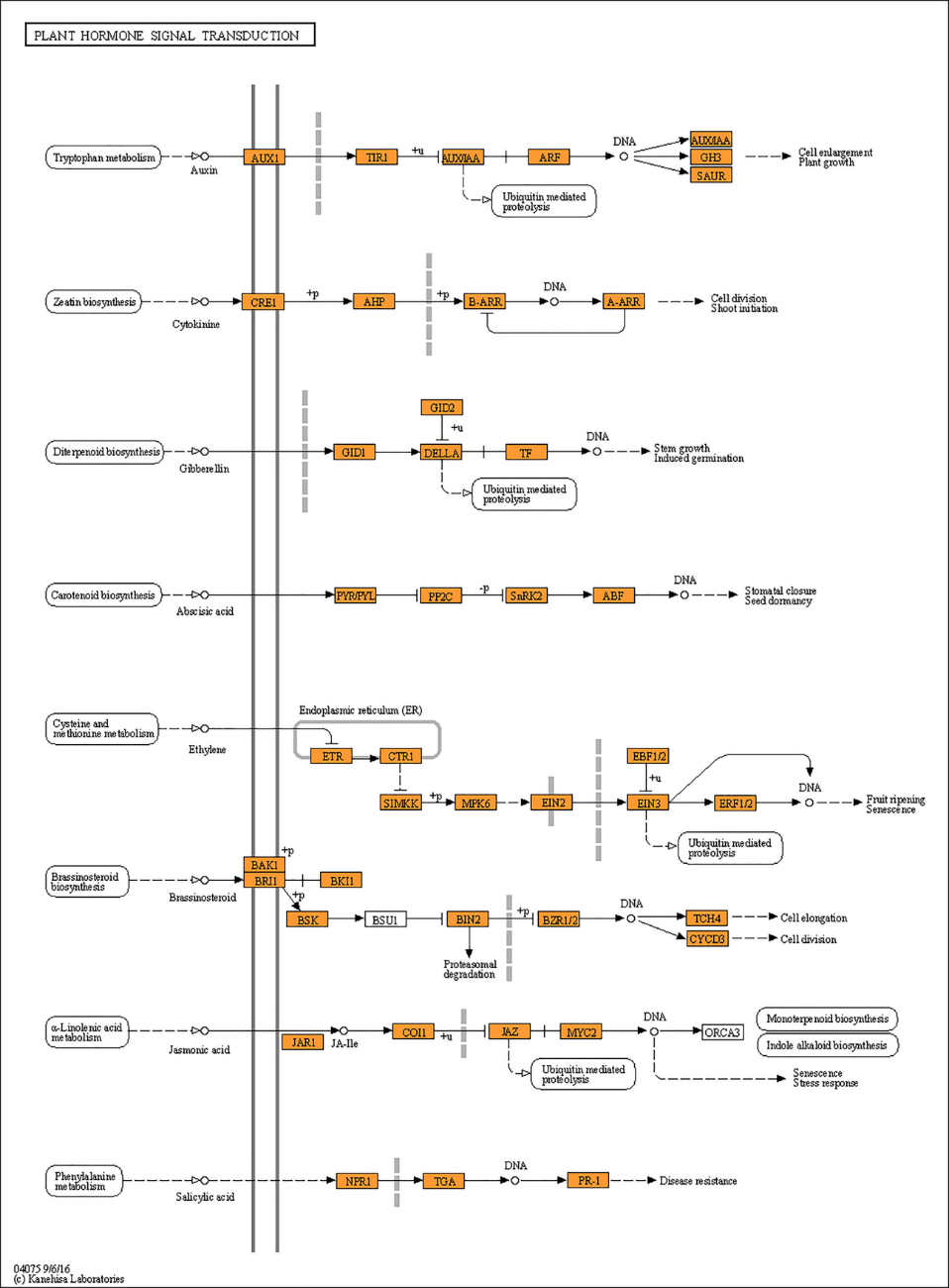


S2 Fig. MAPK- signaling pathway mapped to the KEGG annotation against *A. spathulifolius*.

Supplement: S2 Fig — (DOCX) [file pone.0244132.s002.docx]

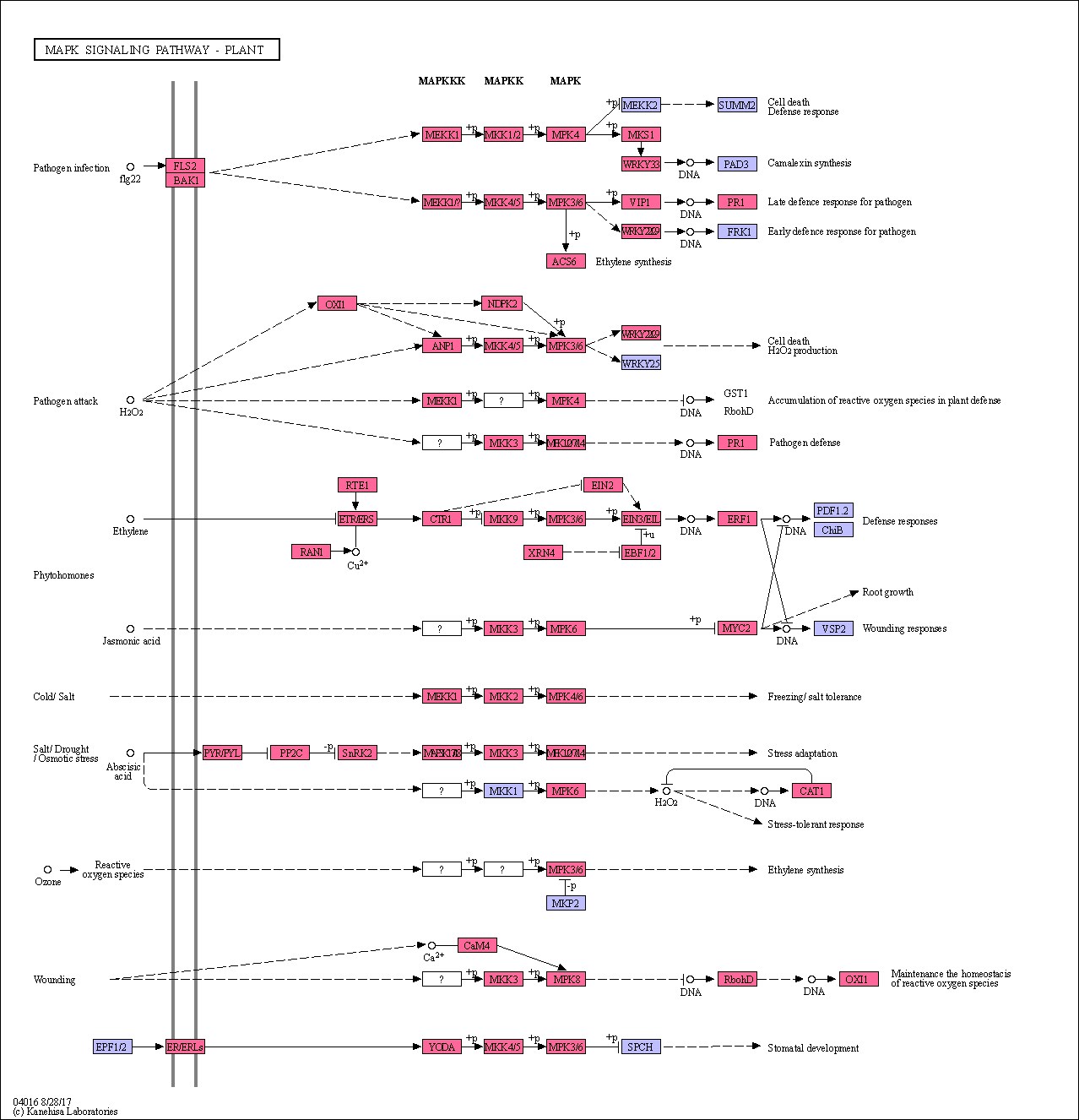


S3 Fig. Plant hormone signaling pathway in *A. spathulifolius* against KEGG annotation.

Supplement: S3 Fig — (DOCX) [file pone.0244132.s003.docx]
