# Supplementary material for: Aster spathulifolius Maxim. a leaf transcriptome provides an overall functional characterization, discovery of SSR marker and phylogeny analysis
Source: PLoS One. 2020 Dec 23;15(12):e0244132. doi: 10.1371/journal.pone.0244132 (PMC7757906; doi:10.1371/journal.pone.0244132)
Supplement: S4 Fig — (DOCX) [file pone.0244132.s004.docx]

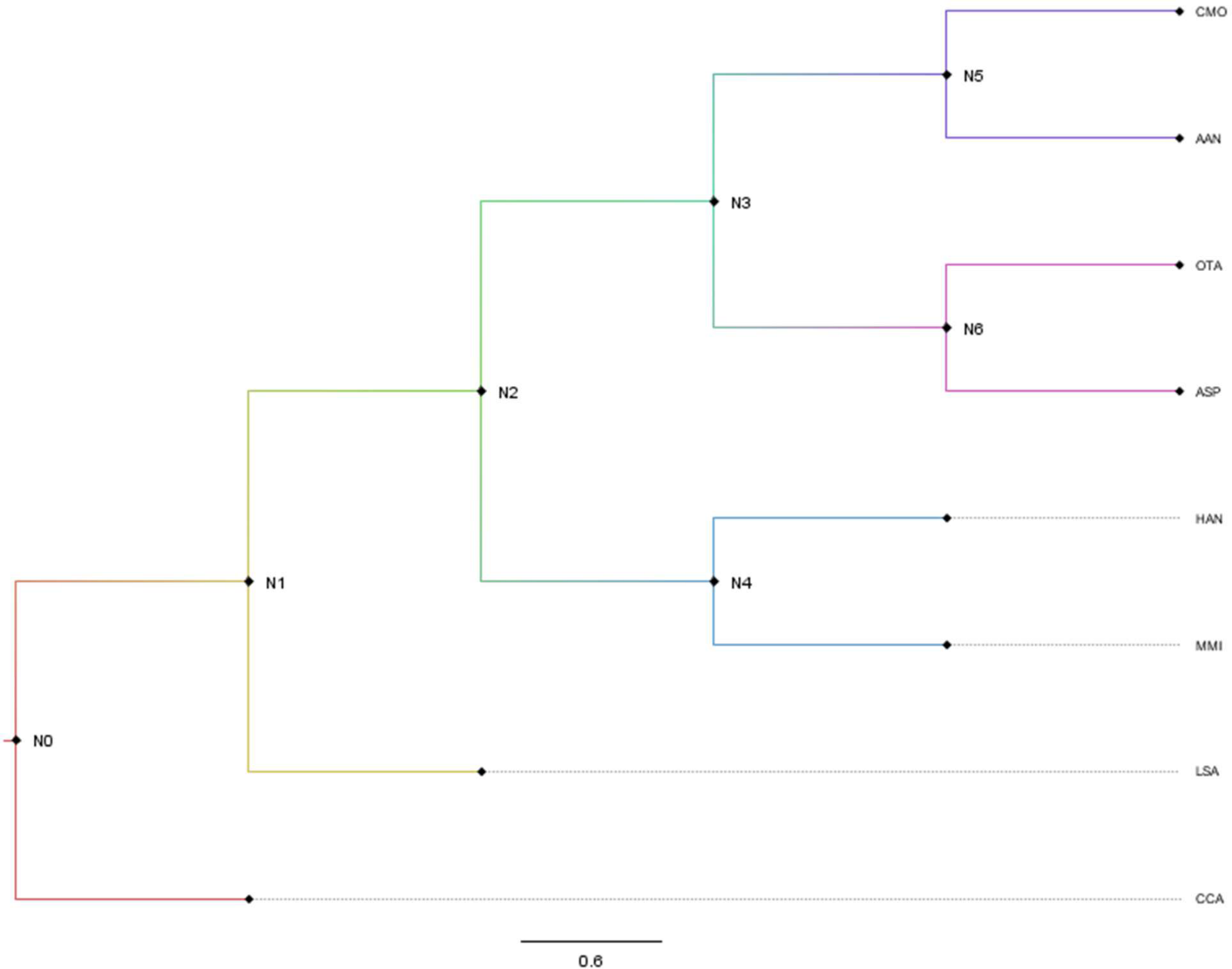


S4 Fig. Orthofinder: gene duplication prediction among the Asteraceae family, N1-N6 indicates the duplication of gene in order, AAN: *A. annua*, OTA: *O. taihangensis*, ASP: *A. spathulifolius*, HAN: *H. annuus*, MMI: *M. micranta*, LSA: *L. sativa*, CCA: *C. cardunculus*.
