# Supplementary material for: Aster spathulifolius Maxim. a leaf transcriptome provides an overall functional characterization, discovery of SSR marker and phylogeny analysis
Source: PLoS One. 2020 Dec 23;15(12):e0244132. doi: 10.1371/journal.pone.0244132 (PMC7757906; doi:10.1371/journal.pone.0244132)
Supplement: S1 Table — (DOCX) [file pone.0244132.s005.docx]

S1 Table. RSEM calculate expression matrix show main gene involved in Phenylpropanoid pathway at FPKM rate.

| transcript_id | Gene | EC number | length | effective_length | expected_count | TPM | FPKM | IsoPct |
| --- | --- | --- | --- | --- | --- | --- | --- | --- |
| TRINITY_DN12273_c0_g1_i1 | cinnamoyl-CoA reductase | EC:1.2.1.44 | 1005 | 810.1 | 1091 | 39204.2 | 21741 | 100 |
| TRINITY_DN12978_c0_g1_i2 | caffeic acid 3-O-methyltransferase | EC:2.1.1.68 | 1074 | 879.1 | 1346 | 44571.09 | 24717.3 | 100 |
| TRINITY_DN1109_c0_g1_i3.p1  TRINITY_DN457_c0_g1_i3.p1  TRINITY_DN1297_c0_g1_i5 | phenylalanine ammonia-lyase | EC:4.3.1.24 | 2154 | 1959.1 | 56575 | 840648.07 | 466189 | 100 |
| TRINITY_DN16870_c0_g1_i4 | shikimate O-hydroxycinnamoyltransferase | EC:2.3.1.133 | 1284 | 1089.1 | 663 | 17721.16 | 9827.43 | 100 |
| TRINITY_DN20381_c0_g1_i1 | 4-coumarate--CoA ligase | EC:6.2.1.12 | 1617 | 1422.1 | 1296 | 26529.02 | 14711.9 | 100 |
| TRINITY_DN38755_c0_g1_i1 | feruloyl-CoA 6-hydroxylase | EC:1.14.11.61 | 1128 | 933.1 | 223 | 6957.02 | 3858.08 | 100 |
| TRINITY_DN4641_c0_g1_i1 | caffeoylshikimate esterase | EC: 3.1.1.- | 1092 | 897.1 | 751 | 24369.44 | 13514.3 | 100 |
