# Supplementary material for: Aster spathulifolius Maxim. a leaf transcriptome provides an overall functional characterization, discovery of SSR marker and phylogeny analysis
Source: PLoS One. 2020 Dec 23;15(12):e0244132. doi: 10.1371/journal.pone.0244132 (PMC7757906; doi:10.1371/journal.pone.0244132)
Supplement: S2 Table — (DOCX) [file pone.0244132.s006.docx]

S2 Table. NCBI plastid and SRA database accession number along with information of assembled unigene in total.

| S.No | Accession number of chloroplast | SRA | Species | genus | family | Total unigene | GC |
| --- | --- | --- | --- | --- | --- | --- | --- |
| 1 | NC_042787.1 | SRR8703844 | *Opisthopappus taihangenesis* | *Opisthopappus* | Asteraceae | 85600 | 40.50% |
| 2 | NC_007977.1 | SRR6061857 | *Helianthus annuna* | *Helianthus* | Asteraceae | 77130 | 40.80% |
| 3 | NC_007578 | SRR6374706 | *Lactuca sativa* | *Lactuca* | Asteraceae | 47883 | 40.34% |
| 4 | KM035764 | SRR403069 | *Cynara cardunculus* | *Cynara* | Asteraceae | 32816 | 42.30% |
| 5 | NC_034683.1 | SRR169031 | *Artemisia annua* | *Artemisia* | Asteraceae | 89384 | 39.13% |
| 6 | NC_031833.1 | SRR6428968 | *Mikania micranta* | *Mikrania* | Asteraceae | 103225 | 41.30% |
| 7 | NC_020092.1 | SRR6933857 | *Chrysanthemum x morifolium* | *Chrysanthemum* | Asteraceae | 59834 | 41.70% |
| 8 | NC_027434.1 | SRR10724565 | *Aster spathulifolius* | *Aster* | Asteraceae | 98660 | 40.53% |
| 9 | NC_040933.1 | SRR7429941 | *Scaevola taccada* | *Scaevola* | Goodeniaceae | 24225 | 40.12% |
